# White matter tract signatures of impaired social cognition in frontotemporal lobar degeneration

**DOI:** 10.1016/j.nicl.2015.06.005

**Published:** 2015-06-23

**Authors:** Laura E. Downey, Colin J. Mahoney, Aisling H. Buckley, Hannah L. Golden, Susie M. Henley, Nicole Schmitz, Jonathan M. Schott, Ivor J. Simpson, Sebastien Ourselin, Nick C. Fox, Sebastian J. Crutch, Jason D. Warren

**Affiliations:** a Dementia Research Centre, UCL Institute of Neurology, University College London, London, UK; b Centre for Medical Image Computing, University College London, London, UK

**Keywords:** Frontotemporal dementia, Social cognition, Sarcasm, Emotion, Diffusion tensor imaging, Voxel based morphometry

## Abstract

Impairments of social cognition are often leading features in frontotemporal lobar degeneration (FTLD) and likely to reflect large-scale brain network disintegration. However, the neuroanatomical basis of impaired social cognition in FTLD and the role of white matter connections have not been defined. Here we assessed social cognition in a cohort of patients representing two core syndromes of FTLD, behavioural variant frontotemporal dementia (bvFTD; n = 29) and semantic variant primary progressive aphasia (svPPA; n = 15), relative to healthy older individuals (n = 37) using two components of the Awareness of Social Inference Test, canonical emotion identification and sarcasm identification. Diffusion tensor imaging (DTI) was used to derive white matter tract correlates of social cognition performance and compared with the distribution of grey matter atrophy on voxel-based morphometry. The bvFTD and svPPA groups showed comparably severe deficits for identification of canonical emotions and sarcasm, and these deficits were correlated with distributed and overlapping white matter tract alterations particularly affecting frontotemporal connections in the right cerebral hemisphere**.** The most robust DTI associations were identified in white matter tracts linking cognitive and evaluative processing with emotional responses: anterior thalamic radiation, fornix (emotion identification) and uncinate fasciculus (sarcasm identification). DTI associations of impaired social cognition were more consistent than corresponding grey matter associations. These findings delineate a brain network substrate for the social impairment that characterises FTLD syndromes. The findings further suggest that DTI can generate sensitive and functionally relevant indexes of white matter damage in FTLD, with potential to transcend conventional syndrome boundaries.

## Highlights

• Social cognition deficits define frontotemporal dementias but are poorly understood.
• We studied brain network correlates of sarcasm processing in these dementias with DTI.
• Sarcasm deficits were particularly linked to right frontotemporal tract changes.
• DTI generates functionally relevant metrics of white matter damage in these dementias.

## 1 Introduction

Frontotemporal lobar degeneration (FTLD) refers to a heterogeneous group of non-Alzheimer dementias collectively characterised by progressive atrophy of the frontal and temporal lobes and presenting with insidious disintegration of social behaviour or language (Ratnavalli et al., 2002; Rosen et al., 2005; Hodges and Patterson, 2007; Kipps et al., 2009; Rohrer and Warren, 2010; Omar et al., 2011; Rascovsky et al., 2011; Gorno-Tempini et al., 2011; Rohrer et al., 2011; Whitwell and Josephs, 2011; McGinnis, 2012; Warren et al., 2013a). These diseases collectively constitute a common cause of young-onset ‘frontotemporal dementia’ (Ratnavalli et al., 2002), pose substantial problems of nosology and diagnosis, and highlight the fundamental neurobiological problem of selective neurodegeneration.

These challenges are well illustrated by the canonical FTLD syndromes of behavioural variant frontotemporal dementia (bvFTD) and the semantic variant of primary progressive aphasia (svPPA, or semantic dementia). Clinically, bvFTD characteristically manifests with progressive behavioural deterioration leading to severe social dysfunction that may be misdiagnosed as a primary psychiatric disorder (Rosen et al., 2005; Kipps et al., 2009; Omar et al., 2011; Rascovsky et al., 2011), while svPPA presents with progressive erosion of semantic memory manifesting as loss of knowledge about words, objects and concepts, typically with supervening behavioural and personality changes later in the course (Hodges and Patterson, 2007; Rohrer and Warren, 2010; Gorno-Tempini et al., 2011). Both syndromes potentially hold unique insights into the neurobiology of social cognition and the impact of disease on its critical brain substrates. Emerging structural and functional neuroimaging evidence has implicated specific large-scale brain networks in the pathogenesis of bvFTD and svPPA: in the case of bvFTD, a ‘salience’ network that processes emotionally significant internal and external stimuli including social signals (Seeley et al., 2009; Zhou et al., 2010) and links distributed brain regions including the prefrontal cortex, orbitofrontal cortex, anterior cingulate, and insula; and in the case of svPPA, a ‘semantic’ network that associates multimodal sensory and symbolic data with meaning, instantiated in the anterior temporal and inferior frontal lobes (Hodges and Patterson, 2007; Fletcher and Warren, 2011). Differential involvement of these networks may provide candidate brain substrates for social cognition deficits in these syndromes (Seeley et al., 2009; Zhou et al., 2010, 2012; Irish et al., 2011; Raj et al., 2012).

Social cognition is a multi-dimensional and still poorly understood aspect of human brain function (Zahn et al., 2007; Adolphs, 2009; Kennedy and Adolphs, 2012; Olson et al., 2013); it typically entails emotional, semantic, mnestic and evaluative processing of sensory signals, and yet specialised brain systems underpinning social cognition have been inferred from evidence in the healthy brain and in disease states, suggesting that it might be a useful paradigm for detecting and tracking the clinical course of diseases in the FTLD spectrum. Indeed, the multi-dimensionality of social cognition is reflected in the diverse deficits described in bvFTD, including multimodal recognition of canonical emotions (Omar et al., 2011; Kumfor et al., 2013), empathic concern and perspective taking (Rankin et al., 2005; Mahoney et al., 2011), mentalising (Le Bouc et al., 2012; Downey et al., 2013), perception of humour and sarcasm (Snowden et al., 2003; Kipps et al., 2009), affective decision making (Torralva et al., 2007), moral reasoning (Chiong et al., 2013) and conceptualising self in relation to others (Irish et al., 2011). Although social cognition deficits in svPPA are less widely documented, a number of studies have reported similar abnormalities of social functioning in these patients (Rankin et al., 2009; Zahn et al., 2009; Duval et al., 2012).

The processing of sarcasm is an attractive model for probing component processes of social cognition that are vulnerable in FTLD syndromes (Kipps et al., 2009; Rankin et al., 2009). Sarcasm exemplifies a familiar and relatively simple paralinguistic cue that must be processed according to social context in order to understand the verbal message; as a stimulus feature, sarcastic intent is straightforward to manipulate and its detection can be reliably assessed (McDonald et al., 2006). Improved understanding of the brain mechanisms of sarcasm processing and social dysfunction more generally could potentially facilitate earlier and more accurate diagnosis and symptom management in FTLD and ultimately, evaluation of therapies in clinical trials. The overlapping phenomenology of interpersonal difficulties exhibited by patients with bvFTD and svPPA might reflect underlying neural substrates that are at least partly shared, consistent with convergent profiles of regional brain damage in these syndromes (McGinnis, 2012; Warren et al., 2013b); and impaired detection of sarcasm has been shown to predict and to track progression in bvFTD (Kipps et al., 2009; Kumfor et al., 2014). Impaired understanding of sarcasm has been linked to damage involving distributed neural networks including the ventro-medial prefrontal cortex, orbitofrontal cortex, anterior temporal lobes and their connections, also implicated in processing sarcasm and other social signals in the healthy brain (Zahn et al., 2007; Adolphs, 2009; Carrington and Bailey, 2009; Kipps et al., 2009; Davis et al., 2015).

This work contributes to a growing body of evidence associating particular social cognition deficits with regional brain damage in FTLD (Mahoney et al., 2011; Le Bouc et al., 2012; Chiong et al., 2013; Rankin et al., 2009; Zahn et al., 2009; Moll et al., 2011). To date, however, neuroanatomical correlative studies have focussed essentially on grey matter alterations: if (as neuroimaging work in the healthy brain strongly suggests), the processes that underpin social cognition are distributed across brain networks, then techniques that can assess structural and functional connections between individual brain regions will be required in order to delineate fully the brain mechanisms that support social cognition and the effects of disease. This is particularly relevant to neurodegenerative diseases such as FTLD that are inherently network-based (Warren et al., 2013b). White matter tracts bind brain networks, and techniques such as diffusion tensor imaging (DTI) can assess the microstructural integrity of white matter connections and correlate these with clinical deficits (Whitwell et al., 2010; Agosta et al., 2012; Mahoney et al., 2013; Tovar-Moll et al., 2014). DTI has been shown to detect white matter alterations in genetically mediated FTLD prior to the onset of symptoms or cortical atrophy, suggesting a potential role as a sensitive disease biomarker (Dopper et al., 2013).

Here we investigated white matter correlates of social cognition impairment in bvFTD and svPPA using DTI. We correlated DTI metrics with indices of canonical emotion identification (a key component of social signal coding) and sarcasm identification (a model of everyday social signal interpretation) in naturalistic vignettes. DTI data were analysed using a tract-based spatial statistics (TBSS) processing pipeline that makes minimal prior assumptions about key sites of disease involvement and is therefore relatively anatomically unbiased. White matter alterations were correlated with the distribution of grey matter atrophy assessed using voxel-based morphometry (VBM). We hypothesised that white matter alterations would target pathways binding the distributed fronto-temporal and limbic networks previously implicated in neuroimaging studies of social cognition in the healthy brain and in neurodegenerative disease. We further hypothesised that white matter signatures of impaired social cognition would constitute ‘trans-syndromic’ substrates for the overlapping behavioural deficits that characterise the bvFTD and svPPA syndromes clinically. DTI measures of axial (AX), radial (RD) and total (trace, TR) diffusivity and fractional anisotropy (FA) were assessed in parallel, given previous work suggesting that different DTI measures may constitute relatively specific indices of structural or functional integrity in white matter pathways (Acosta-Cabronero et al., 2010; Whitwell et al., 2010; Agosta et al., 2012; Mahoney et al., 2013, 2014).

## 2 Methods

### 2.1 Participants

Twenty-nine patients fulfilling consensus criteria for probable or definite bvFTD (Rascovsky et al., 2011) and 15 patients fulfilling consensus criteria for svPPA (Gorno-Tempini et al., 2011) were recruited from a specialist cognitive disorders clinic (details summarised in Table 1). Thirty-seven healthy older individuals with no history of neurological or psychiatric illness also participated. All participants underwent a structured clinical evaluation and an assessment of general neuropsychological functions covering general intellect, memory, semantic, linguistic, executive and perceptual domains (Table 1; see also Supplementary material on-line). Informed consent was obtained for all participants and the study was approved by the local Research Ethics Committee under Declaration of Helsinki guidelines.

### 2.2 Assessment of social cognition

The Awareness of Social Inference Test (TASIT) (McDonald et al., 2006) was used to assess participants' ability to identify basic emotions and sarcastic intent in social situations. This test has been widely used as a measure of social cognition performance in clinical contexts and requires interpretation of posed but relatively naturalistic social scenarios. We administered an abbreviated TASIT comprising emotion identification and sarcasm identification subtests derived from the first portions of the respective subtests in the full TASIT. In the emotion identification subtest, 14 audio-visual video vignettes (each 15–20 s duration) conveying either a positive (surprised, happy), neutral or negative (anger, disgust, sadness or anxiety) emotional valence were presented in randomised order; the task on each trial was to decide which emotion was dominantly portrayed in a seven-alternative forced-choice procedure. In the sarcasm identification subtest, nine video vignettes were presented in randomised order, each displaying one of three conditions: sincere intent, simple sarcasm (i.e., intended meaning simply at odds with the message surface structure as delivered) or paradoxical sarcasm (i.e., an elaborated message that would seem nonsensical unless sarcastic intent is recognised, requiring deeper encoding of the social interaction. The task on each trial was to decide how those in the vignette felt, how they were trying to make others feel, and the underlying intent of their interactions or statements (assessed using four questions, yielding a total score of 12 for each condition). Participants were familiarised with each task to ensure that they understood it prior to commencing the test; during the test, no feedback about performance was given and no time limit was imposed. As the TASIT includes a component of verbal comprehension, a trial vignette was administered prior to formal testing to ensure that participants adequately understood the task and the vocabulary used (three more severely affected patients with svPPA were excluded on this basis).

Social cognition measures, general neuropsychological data and general demographic characteristics (age, gender) were compared between participant groups using Stata12©. Repeated-measures analysis of variance regression models were used to assess group differences in performance scores, age and gender. British Picture Vocabulary Scale (BPVS; a standard measure of verbal semantic function, Dunn et al., 1982) scores were included as a covariate in regression models assessing TASIT subtest performance, in order to adjust for verbal comprehension ability.

### 2.3 MRI acquisition

Brain MR images were acquired on a Siemens Trio 3 T MRI scanner using a 32-channel phased array head-coil (Siemens, Erlangen, Germany). Two 64-direction DTI sequences were acquired with a single shot, spin-echo echo planar imaging sequence (55 contiguous axial 2.5 mm slices with 240 mm field of view and 96 × 96 matrix, yielding 2.5 mm isotropic voxels; repetition time: 6800 ms; echo time: 91 ms; b value: 1000 s/mm^2^), augmented with parallel imaging acceleration to reduce susceptibility artefact. Nine sequences without diffusion weighting were also acquired (b = 0 s/mm^2^). Multiple diffusion and non-diffusion weighted scans were acquired to improve signal to noise and to provide multiple independent observations, improving the fit of the tensor model and robustness of the data. In addition a sagittal 3-D magnetisation-prepared rapid gradient echo T1-weighted volumetric MRI (echo time/repetition time/inversion time = 2.9/2200/900 ms, dimensions 256 × 256 × 208, voxel size 1.1 × 1.1 × 1.1 mm) and a coronal fluid-attenuated inversion recovery (FLAIR) MRI were acquired. For all participants, volumetric MRI, DTI and FLAIR sequences were assessed visually in all planes to ensure adequate coverage and to exclude artefacts, unexpected pathology or significant motion.

### 2.4 Image pre-processing

Raw diffusion weighted images were affine-aligned to the first corresponding b0 image using a linear image registration tool (FLIRTv5.5) within the FMRIB Software Library (FSLv4.1.5), in order to correct for motion and eddy currents. The weighting vectors were adjusted to correct for any rigid body motion. DTI volumes were then combined for tensor fitting using Camino (http://cmic.cs.ucl.ac.uk/camino) and FA and other DTI metrics at each voxel derived from the tensor eigenvalues (λ1, λ2, and λ3; where AX = λ1; RD = (λ2 + λ3)/2), TR = (λ1 + λ2 + λ3) and FA is given by:

$$\sqrt{\frac{3\sum_{i=1}^{3}{\left(\lambda_{i}-TR\right)}^{2}}{2\sum_{i=1}^{3}\lambda_{i}{}^{2.}}}$$

To allow group level analysis of DTI metric data, following tensor fitting, images were further processed using the previously published TBSS pipeline (TBSSv1.1) (Smith et al., 2006).

Pre-processing of volumetric MR brain images was performed using the New Segment and DARTEL toolboxes of SPM8 (http://www.fil.ion.ucl.ac.uk/spm) running under Matlab 7.14®. Pre-processing steps were carried out in accordance with current guidelines (Ridgway et al., 2008). Normalisation, segmentation, modulation and smoothing of grey matter images using a 6 mm kernel size were performed using default parameter settings and the final images were affine-transformed into MNI space prior to analysis. In order to adjust for individual differences in head size total intracranial volume (TIV) was calculated for each participant by summing grey matter, white matter and cerebrospinal fluid volumes following segmentation of all three tissue classes, and used as a covariate in subsequent analyses. In order to display results, a study-specific template brain image was created by warping all native-space whole-brain images to the final DARTEL template and calculating the average of the warped brain images.

### 2.5 Analyses of neuroimaging data

General linear models were used to examine the relationship between social cognition performance measures and both white matter tract microstructure and regional grey matter volume. Separate models were used to assess TASIT emotion identification scores and TASIT total sarcasm identification, simple sarcasm identification and paradoxical sarcasm identification scores as functions of regional grey matter volume and diffusivity metrics, with age, TIV (calculated as described above) and disease group membership included as covariates. To minimise confounding effects from correlated general semantic, executive or disease severity factors, raw scores on the BPVS and a Stroop ink colour naming test (a standard executive measure) were included as additional nuisance covariates in the model design. In the DTI analyses, FA, AX, RD and TR metrics were analysed separately. In each case, relationships between the behavioural and neuroimaging metrics were assessed across the two syndromic groups, in each syndromic group separately, and contrasting the two syndromic groups. In the grey matter analysis an additional step was applied to minimise voxel drop-out due to marked local regional atrophy in particular scans. The analysis was performed within a customised brain mask based on a specified voxel threshold intensity criterion (Ridgway et al., 2009) whereby a voxel was included in the analysis if grey matter intensity at that voxel was >0.1 in >70% of the participants.

Statistical analyses for both white matter and grey matter analyses were implemented using the permutation-based (non-parametric) randomise tool within FSL with 5000 permutations generated for each test. For all analyses, a significance threshold (p = 0.05) was applied following correction for multiple comparisons using family-wise error correction with threshold-free cluster enhancement (Smith and Nichols, 2009) over the whole brain. Further information on the significant results generated from each model in the DTI analysis was extracted using FSL's ‘*cluster*’ to determine if multiple anatomical clusters of white matter alteration occurred within each contrast and to extract information on the statistical significance and spatial extent of each anatomical cluster. To determine involvement of specific white matter tracts the JHU ICBM-DTI-81 DTI white matter atlas was registered to the study specific mean FA image (Wakana et al., 2007). The peak co-ordinates from each cluster were located using the atlas to determine if they lay within the boundary of a particular white matter tract; if the peak co-ordinates were ‘unclassifiable’, then the co-ordinates were inspected visually to assign an approximate location of the cluster within a particular brain region.

Maps of disease-associated grey and white matter alteration were generated for each syndromic group relative to a historical age- and gender-matched cohort of healthy individuals (n = 20, seven female, mean age 64.5 ± 4.5 years) for which brain MR images were previously obtained using the same scanner and acquisition parameters. Grey and white matter disease maps were generated using the same parameters as previously specified.

## 3 Results

### 3.1 General characteristics of participants

General characteristics of participants are summarised in Table 1. The patient and healthy control groups were well matched for age (p = 0.74) and patient groups had similar disease durations (p = 0.86). Male participants were over-represented in the bvFTD group (p = 0.009) compared to healthy controls. The bvFTD and svPPA groups each showed the anticipated profile of cognitive deficits relative to the healthy control group and to the other syndromic group (see Table 1). The svPPA group showed significantly worse performance on measures of single word comprehension, naming and vocabulary (all p < 0.05; see Table 1). All patients with bvFTD had MRI evidence of fronto-temporal lobar atrophy and all patients with svPPA had asymmetric (predominantly left-sided) anterior inferior temporal lobe atrophy. No patient had radiological evidence of a substantial concomitant vascular burden.

### 3.2 Social cognition performance

Social cognition data are summarised in Table 1. Compared with healthy controls both the bvFTD and svPPA groups showed significant deficits in TASIT emotion identification (p < 0.001), simple sarcasm identification (p < 0.001), paradoxical sarcasm identification (p < 0.01) and combined (total) sarcasm identification (p < 0.05). There were no significant performance differences between the patient groups for emotion identification, or identification of simple or paradoxical sarcasm.

### 3.3 Grey matter and white matter disease maps

As anticipated, compared with healthy controls both syndromic groups showed extensive profiles of grey matter atrophy and changes in cerebral white matter integrity. The bvFTD group showed distributed bi-hemispheric atrophy involving the anterior temporal lobes, mesial temporal structures, insular, prefrontal and orbitofrontal cortices; while the svPPA group showed an overlapping but distinctive profile of bi-hemispheric atrophy involving the anterior inferior temporal lobes (more marked on the left) extending into the posterior inferior temporal and orbitofrontal cortices (see Fig. S1 in Supplementary material on-line). White matter alterations were evident bi-hemispherically but with a fronto-temporal gradient (see Fig. S2 in Supplementary material on-line). The bvFTD group had both dorsal and ventral white matter alterations, most extensive in the bilateral uncinate fasciculus, corpus callosum and cingulum bundle, with less marked changes posteriorly including the parieto-occipital inferior longitudinal fasciculus. The svPPA group had a more ventral profile of white matter alterations most prominent in the uncinate fasciculus bilaterally and also involving the corpus callosum and bilateral cingulum bundle.

### 3.4 White matter tract associations of social cognition performance

**Fig. 1:**
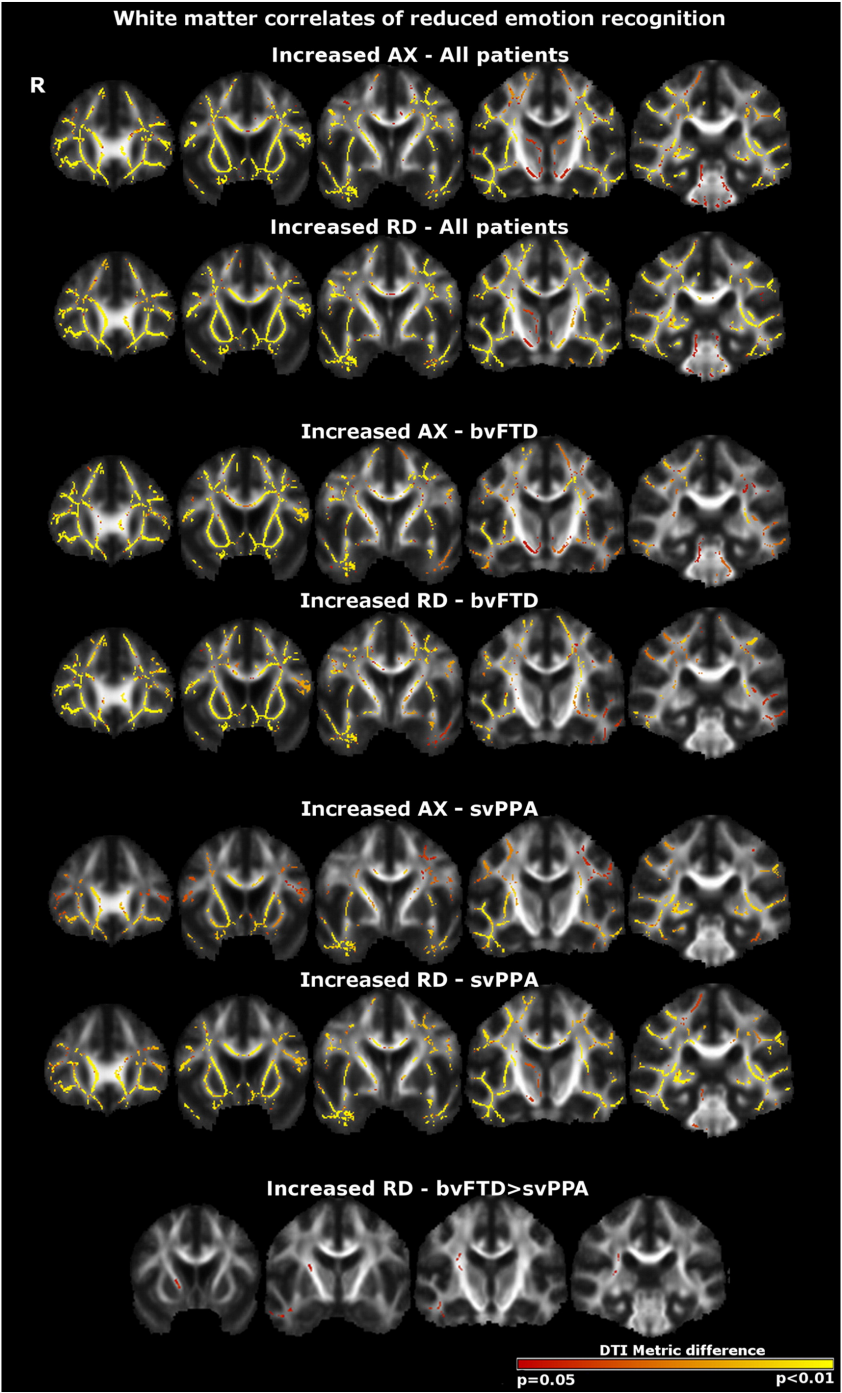
White matter correlates of reduced emotion identification performance in both patient groups (top panels), the bvFTD group alone (middle panels) and the svPPA group alone (bottom panels). Results are overlaid on a customised group template brain image and displayed in MNI standard space; the right hemisphere (R) is displayed on the left. The colour scale indexes p-value after family-wise error correction over the whole brain at p < 0.05. Key: AX, axial diffusivity; bvFTD, behavioural variant frontotemporal dementia; RD, radial diffusivity, svPPA, semantic variant of primary progressive aphasia.

**Fig. 2:**
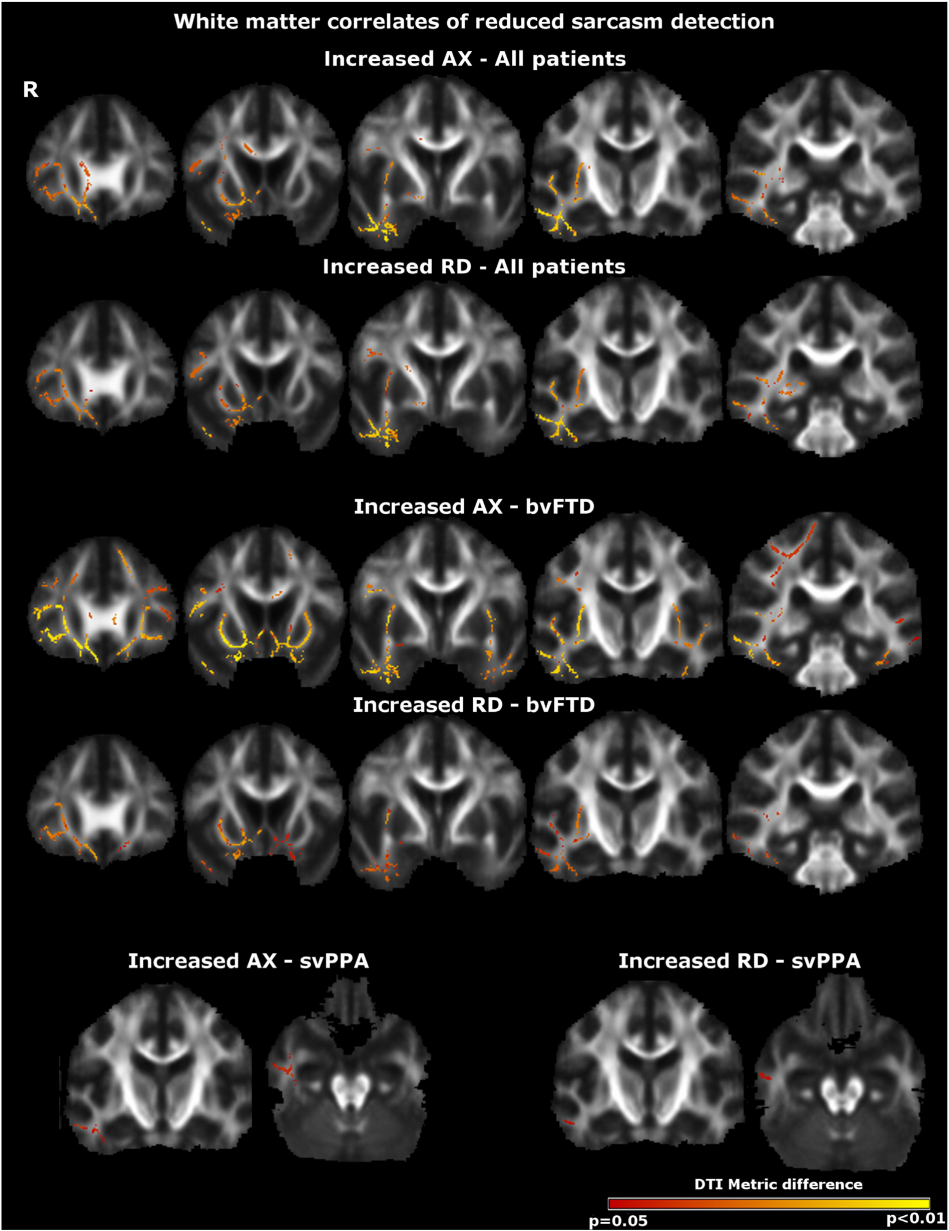
White matter tract correlates of reduced sarcasm identification across both patient groups (top panels), in the bvFTD group alone (middle panels) and in the svPPA group alone (bottom panels). Results are overlaid on a customised group template brain image and displayed in MNI standard space; the right hemisphere (R) is displayed on the left. The colour scale indexes p-value after family-wise error correction over the whole brain at p < 0.05. Key: AX, axial diffusivity; bvFTD, behavioural variant frontotemporal dementia; RD, radial diffusivity, svPPA, semantic variant of primary progressive aphasia.

Maps of DTI metric alterations associated with performance on social cognition tasks are shown in Figs. 1 and 2; data for peak co-ordinates and clusters of white matter alterations associated with task performance are summarised in Tables 2 and 3. All results are reported thresholded at p = 0.05 after family-wise error correction for multiple comparisons over the whole brain.

Across both patient groups, TASIT emotion identification score was inversely correlated with AX, RD and TR and positively correlated with FA extensively over the dorsal, ventral and commissural white matter tracts in both cerebral hemispheres (Figs. 1 and 2); the largest effects in terms of statistical significance and extent (Table 2) were demonstrated in the frontal subcortical projection pathways (right anterior thalamic radiation) and fornix. Within the bvFTD group alone, emotion identification impairment was associated with white matter alterations predominantly in the corpus callosum and fornix. Within the svPPA group alone, emotion identification score was inversely correlated with increased AX and RD predominantly in the right anterior thalamic radiation. Comparing syndromic groups, the inverse correlation between total emotion score and increased RD was significantly stronger in the right anterior thalamic radiations and right inferior longitudinal fasciculus in the svPPA group than the bvFTD group; the reverse contrast did not identify any significant white matter associations.

Across both patient groups, TASIT total sarcasm (simple sarcasm + paradoxical sarcasm) identification score was inversely correlated with AX, RD and TR in bi-hemispheric but predominantly right temporal and inferior frontal white matter tracts; the largest effects in terms of statistical significance and extent (Table 3) were demonstrated in the right uncinate fasciculus (Fig. 2). Within the bvFTD group, sarcasm identification score correlated with predominantly right-sided but bilateral temporal and inferior frontal white matter alterations; largest effects were demonstrated in the right uncinate fasciculus and right anterior thalamic radiation. Within the svPPA group, sarcasm identification score correlated with more discrete right temporal white matter alterations; the largest effects were demonstrated in the right inferior longitudinal fasciculus. Contrasts regressing total simple sarcasm score and total paradoxical sarcasm score did not identify any additional significant white matter associations. Comparisons between syndromic groups did not identify any significant differential white matter associations.

Across both syndromic groups (Tables 2 and 3), white matter correlates of emotion identification and sarcasm identification were signalled by AX, RD and TR alterations, with partial convergence among these metrics. However, the signal with FA was much less extensive and less consistent.

### 3.5 Grey matter associations of social cognition performance

**Fig. 3:**
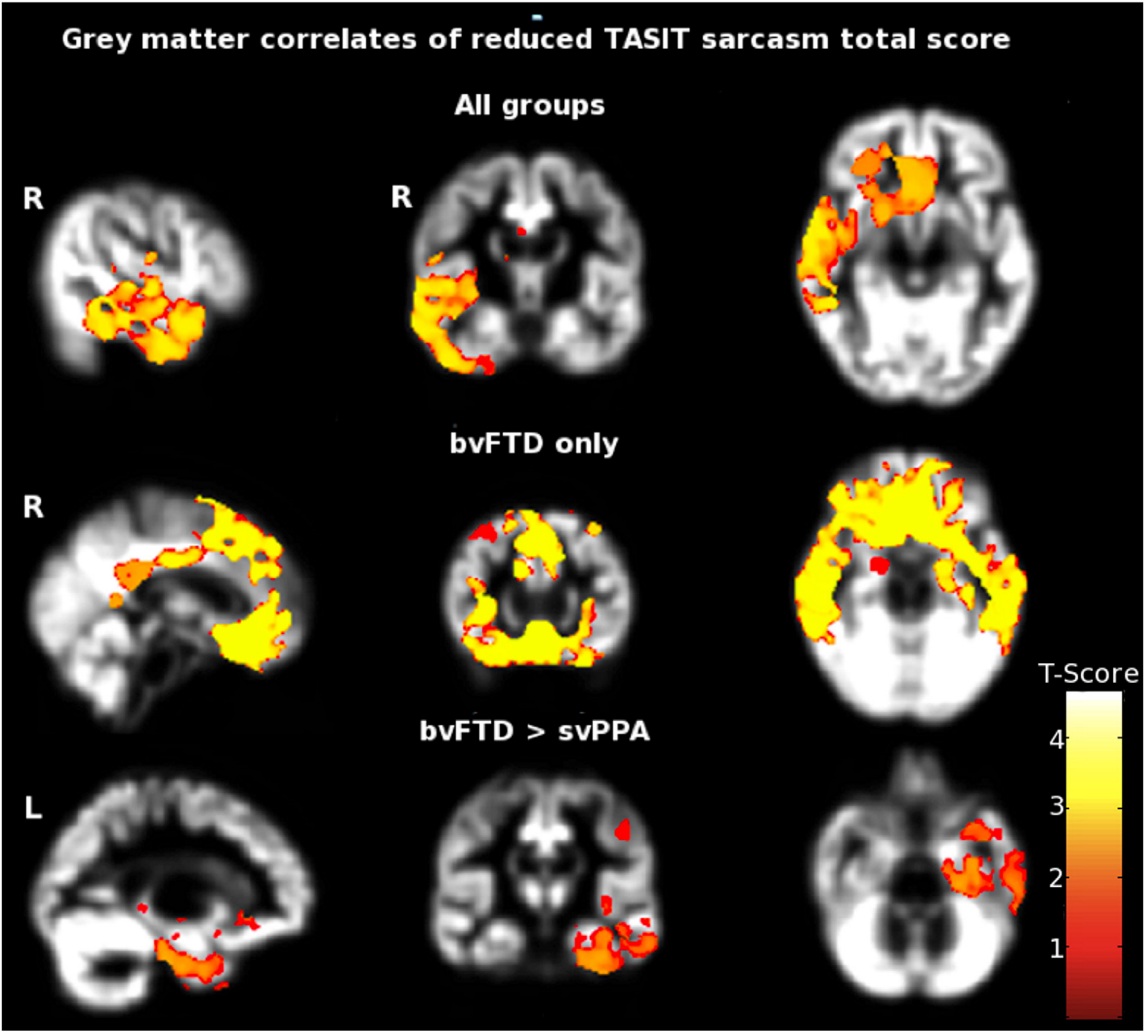
Grey matter correlates of sarcasm identification across both bvFTD and svPPA participant groups. Results are overlaid on a customised group template brain image and displayed in MNI standard space; the right hemisphere is displayed on the left in coronal and axial sections and sagittal sections of right (R) and left (L) cerebral hemispheres are indicated. The colour scale indexes t score after family-wise error correction over the whole brain at p < 0.05. Key: bvFTD, behavioural variant frontotemporal dementia; svPPA, semantic variant of primary progressive aphasia; TASIT, the Awareness of Social Inference Test.

Statistical parametric maps of regional grey matter volume associated with performance on social cognition tasks are shown in Fig. 3; local maxima and clusters of regional grey matter voxels associated with social cognition performance are summarised in Table 4, at the same corrected threshold (p = 0.05) adopted for DTI data. Across both patient groups, TASIT total sarcasm identification score was positively correlated with regional grey matter in the right anterior temporal lobe and orbitofrontal cortex. Within the bvFTD group alone, TASIT total sarcasm identification score was correlated with regional grey matter volume in the bilateral orbitofrontal cortex and anterior temporal lobes. No significant grey matter correlations with sarcasm identification were observed in the svPPA group alone. Comparing disease groups, TASIT total sarcasm identification score was significantly more strongly correlated with grey matter in the left parahippocampal and fusiform gyri in the bvFTD group than the svPPA group. There were no significant grey matter correlations with performance on the TASIT emotion identification task.

These grey matter correlates of social cognition performance from the VBM analysis were only partly convergent with the white matter correlates identified in the DTI analysis (Figs. 1–3). Convergence between the grey and white matter modalities was most clearly shown in the regional emphasis of the changes correlated with sarcasm identification in the right anterior temporal and inferior frontal lobes. In contrast, no grey matter correlates of emotion identification were identified to set beside the white matter correlates at the prescribed significance threshold.

## 4 Discussion

Here we have used an anatomically unbiased DTI protocol to delineate white matter tract associations of cognitive processes relevant to social signal encoding (canonical emotion identification) and interpretation (sarcasm identification) in two canonical FTLD syndromes with prominent social difficulties. In line with previous work (Torralva et al., 2007; Kipps et al., 2009; Rankin et al., 2009; Duval et al., 2012; Shany-Ur et al., 2012), both the bvFTD and svPPA groups exhibited deficits of emotion identification and sarcasm interpretation and deficits were comparably severe in both groups. White matter signatures of brain network damage underpinning these social cognition deficits were widely distributed, overlapping networks implicated in social cognition in the healthy brain (Adolphs, 2009; Kennedy and Adolphs, 2012; Zahn et al., 2009; Carrington and Bailey, 2009) and in association with focal brain damage (Rankin et al., 2005, 2009; Mahoney et al., 2011; Moll et al., 2011; Downey et al., 2013). Furthermore, there was extensive overlap of white matter tract signatures between the bvFTD and svPPA groups: the evidence for separable, syndrome-specific signatures (i.e., profiles of neuroanatomical associations that differed between syndromes) was relatively sparse, consistent with core white matter tract-based mechanisms of social cognitive dysfunction that are common to both bvFTD and svPPA.

White matter correlates of emotion and sarcasm processing here were identified after adjusting for generic cognitive (semantic and executive) indexes, suggesting a relatively specific association with social cognitive operations rather than a more general association with disease severity. Attempts to define neuroanatomical correlates of behaviour in disease states are potentially biased by the brain topography of particular diseases, which tend to limit the associations that can be observed. In the present study, this was addressed by adjusting DTI regression analyses for disease group membership, allowing us to identify associations extending across syndromes in the combined patient cohort as well as syndrome-specific associations. Together this evidence suggests that bvFTD and svPPA involve common large-scale networks mediating social cognition, albeit with a regional emphasis that is modulated by syndrome; and that social cognition processes may constitute ‘trans-syndromic’ signatures of brain dysfunction in FTLD. Moreover, white matter tract correlates of social cognition impairment here were only partly convergent with (and more consistent than) the grey matter correlates identified in the corresponding VBM analyses: this underlines both the potential of DTI to reveal disease signatures that may not be fully delineated using grey matter imaging techniques, and the critical role of white matter pathway integrity in maintaining normal brain network function.

The most robust white matter associations of emotion and sarcasm processing here were identified within tracts previously implicated in linking cognitive and evaluative processing with emotional responses, as part of a broad repertoire of cognitive operations supported by these tracts. Abnormal diffusivity in the anterior thalamic radiation was identified as a correlate of emotion identification in the combined group and within the svPPA group; and a correlate of sarcasm identification within the bvFTD group. The anterior thalamic radiation participates in thalamo–fronto–striatal re-entrant circuits and has widespread projections to the prefrontal cortex and basal forebrain regions. Both in the healthy brain and in a range of neuropsychiatric disorders, this tract has been implicated in various cognitive operations that mediate the social context of emotional signals, including reward and punishment potential, gating and cognitive meaning of affective signals and pervasive induced mood states that may promote evaluation of emotional experiences (Haxby et al., 2002; McIntosh et al., 2008; Cheon et al., 2011; Joutsa et al., 2011; Coenen et al., 2012; Rigucci et al., 2013; Erpelding and Davis, 2013; Fujino et al., 2014; Han et al., 2013; Ameis and Catani, 2015).

Impaired emotion identification in the combined cohort and within the bvFTD group was strongly associated with altered diffusivity in the fornix, consistent with other work in FTLD syndromes (Omar et al., 2011; Kumfor et al., 2013). The fornix is a core limbic tract that links primitive affective, autonomic and homeostatic mechanisms with autobiographical memories and cortical evaluative mechanisms. The role of the fornix is best established within the domain of episodic memory (Rolls, 2015); its contribution to the processing of emotion is more controversial and has been more widely studied in animal models (Decker et al., 1995; Degroot and Treit, 2004; Acevedo et al., 2012). However, in line with the present findings, fornix damage has previously been associated with altered hedonic valence of sensory stimuli and abnormal emotional behaviours (Kazlouski et al., 2011; Maier-Hein et al., 2013; Modi et al., 2013; Poreh et al., 2013; Ameis and Catani, 2015).

Impaired sarcasm detection in the combined cohort and within the bvFTD group was strongly associated with abnormal diffusivity in the uncinate fasciculus: this tract is part of the ‘extended limbic system’ (Pugliese et al., 2009) and plays a key role in associating linguistic and paralinguistic information coded in the anterior superior temporal cortices with affective, motivational, evaluative and mentalising mechanisms in the inferior frontal cortices (Von der Heide et al., 2013; Ameis and Catani, 2015). The uncinate fasciculus has been identified previously as a key locus of pathology in DTI studies of bvFTD and svPPA (Agosta et al., 2012; Mahoney et al., 2013; Tovar-Moll et al., 2014) and damage involving this tract has been associated with altered social behaviour, abnormal evaluation of affective states and impaired empathy (Pugliese et al., 2009; Phan et al., 2009; Tartaglia et al., 2012; Oishi et al., 2015).

The grey matter (VBM) and white matter (DTI) correlates identified here together support previous evidence implicating the right temporal lobe in sarcasm detection (Kipps et al., 2009; Rankin et al., 2009) and further define the brain network underpinning the processing of sarcasm. In particular, the present data are consistent with a model in which the anterior temporal lobe structures process associative meaning and affective tone of speech signals, the inferior frontal cortices disambiguate paralinguistic intent and the uncinate fasciculus acts as the key route of reciprocal information transfer between these grey matter ‘hubs’ (Von der Heide et al., 2013). However, white matter associations of social cognitive performance were not restricted to the anterior frontotemporal tracts: diffusivity correlates were also identified within long intra-hemispheric pathways including the inferior and superior longitudinal and fronto-occipital fasciculi. These pathways have been correlated previously with general measures of social cognition such as ‘emotional intelligence’ (Takeuchi et al., 2013), and may link sensory processing mechanisms with limbic and motor output mechanisms including those that mediate social ‘mirroring’ actions (Hecht et al., 2013). Such long tract associations underline the distributed nature of the brain networks that support social cognition processes (Phan et al., 2009; Mahoney et al., 2011; Kennedy and Adolphs, 2012; Kumfor et al., 2013; Chiong et al., 2013; Ameis and Catani, 2015). More particularly, damage involving the sagittal stratum (a fibre bundle that subsumes a number of long projection pathways) has been linked specifically to impaired sarcasm processing after stroke (Davis et al., 2015).

Of note, primary correlates of social cognition here did not include fronto-insular connections within the salience network previously implicated as central both to the pathogenesis of bvFTD and to processes supporting human social cognition (Seeley et al., 2009; Zhou et al., 2010). The present data suggest that additional, specific pathways and networks may be critical in supporting social cognition in FTLD as the core network disintegrates, while providing potential sites of anatomical convergence (such as the orbitofrontal cortex) where these networks might interact. A role for abnormal network interactions would be consistent with other work in FTLD syndromes (Chiong et al., 2013), while also allowing that extensive white matter pathway damage in other diseases (such as Alzheimer's disease) may leave social capacities relatively unscathed (Acosta-Cabronero et al., 2010).

Our findings illustrate the potential value of DTI as a functional and disease metric in FTLD. Neuroanatomical data derived from DTI and VBM should be compared with caution, given the different properties and technical bases of these modalities; with these caveats in mind, we analysed the present DTI and VBM data in a common pre-processing and statistical framework. Considering both social cognition metrics here, white matter associations were extensive and more consistent than regional grey matter associations, in line with other neuroimaging and neuropathological evidence for extensive white matter pathology in FTLD syndromes (Neumann et al., 2007; Hiji et al., 2008; Whitwell et al., 2010; Agosta et al., 2012; Galantucci et al., 2011; Mahoney et al., 2013). The data corroborate previous work suggesting that DTI may generate sensitive, clinically relevant biomarkers of FTLD syndromes with potential to lead grey matter metrics (Borroni et al., 2007).

White matter signatures based on the FA metric were substantially less extensive than for other diffusivity metrics here (Tables 2 and 3). Information about the specific sensitivities and specificities of particular DTI metrics in disease states and as correlates of clinical dysfunction remains very limited. However, previous work has suggested that FTLD and other neurodegenerative conditions may produce more extensive changes in measures of absolute diffusion, such as RD than ratios such as FA (Acosta-Cabronero et al., 2010; Whitwell et al., 2010; Agosta et al., 2012; Mahoney et al., 2013, 2014). This may in part reflect tissue microarchitectural specificity; AX is likely preferentially to index axonal degeneration and RD, demyelination (Song et al., 2002) in white matter pathways. The present data suggest that certain DTI metrics such as AX and RD may be more suitable than FA for tracking functionally relevant white matter alterations in neurodegenerative syndromes. Also in line with previous evidence (Whitwell et al., 2010, 2011; Rohrer et al., 2011; Mahoney et al., 2013; McMillan et al., 2013), these data raise the further interesting possibility that DTI signatures may have molecular specificity: svPPA is closely associated with TDP-43 pathology and produced a relatively discrete white matter network signature here, whereas bvFTD is pathologically heterogeneous and associated with more widespread network alterations.

Taken together, these findings further define neurobiological signatures for the social impairment that characterises FTLD syndromes, grounded in the emerging neural network paradigm of neurodegenerative disease (Warren et al., 2013b). The findings suggest that certain DTI metrics provide sensitive and functionally relevant indexes of white matter damage in FTLD and support the further assessment of sarcasm as a useful model for probing social and other cognitive functions that depend on large-scale brain networks. It is important to emphasise that the white matter tracts associated with social cognitive impairment here have been implicated in a diverse spectrum of cognitive functions in previous work (Haxby et al., 2002; Poreh et al., 2006; Tartaglia et al., 2012; Han et al., 2013; Von der Heide et al., 2013). Accordingly, we do not argue that involvement of these tracts is a specific harbinger of social cognitive deficits; rather, degeneration of these pathways is likely to contribute to a range of behavioural and cognitive deficits in these syndromes. With that caveat in mind, social signals such as sarcasm may be a particularly sensitive probe of tract function in neurodegenerative diseases, due to the heavy demands such signals impose on the brain networks that decode sensory data for integration with affective, evaluative and mnestic processes.

The present study has several limitations that suggest directions for future work. The findings should be corroborated in larger cohort studies comparing other neurodegenerative diseases and mimic syndromes such as autism, schizophrenia and other primary psychiatric disorders (McIntosh et al., 2008; Miyata et al., 2010; Kazlouski et al., 2011; Rigucci et al., 2013; Fujino et al., 2014; Maier-Hein et al., 2014; Ames and Catani, 2015), which may also produce characteristic white matter changes and for which differentiating biomarkers are particularly required (Niida et al., 2013). These various disorders are likely to provide complementary insights into the neurobiological mechanisms underlying social cognition and dysfunction; in particular, potential parallels between the neural substrates of FTLD and autism are intriguing and should be further explored (Noriuchi et al., 2010; Cheon et al., 2011; Ameis and Catani, 2015). DTI metrics generated using techniques based on whole-brain analysis (as here) lack fine-grained anatomical specificity and are subject to technical issues such as limited resolution of crossing pathways; future tractographic studies targeting specific white matter pathways are needed to address this issue and to define tract anatomy in detail. Longitudinal studies will be essential to establish the sequence of alterations in candidate behavioural and neuroimaging biomarkers, ideally including pre-symptomatic individuals with genetic forms of FTLD, in order to capture very early disease effects and to track disease evolution (Kumfor et al., 2014). Subsequent histopathological correlation will be required to assess the molecular specificity of biomarker signatures (Rohrer et al., 2011; Whitwell and Josephs, 2011; Warren et al., 2013a,b). In the face of these challenges, the present work suggests that white matter metrics of complex behavioural deficits can yield robust signatures of brain network disintegration in FTLD that may transcend conventional clinical and imaging markers.

## Figures and Tables

**Table 1:** Demographic and neuropsychological characteristics of patient and healthy control groups.

	bvFTD	svPPA	Healthy controls
General characteristics			
No.	29	15	37
Age (yrs)	64 (7.1)	65 (6.6)	63 (7.8)
Sex (F:M)	4:25	5:10	19:18
Symptom duration (years)	7.8 (5.3)	6.2 (1.9)	n/a
Neuropsychological assessment			
IQ			
WASI vocab (/80)	**40 (24)**	**26 (24)**^b^	71 (5.3)
WASI blocks (/48)	**23 (19)**	**29 (19)**	46 (11.2)
WASI similarities (/71)	**22 (13)**	**16 (16)**	46 (6.0)
WASI matrices (/32)	**15 (8.5)**	**18 (8.7)**	27 (7.5)
NART (/50)	**26 (15)**	**26 (16)**	42 (5.9)
Episodic memory			
RMT words (/50)	**35 (10)**	**33 (6.0)**	48 (2.6)
RMT faces (/50)	**35 (8.0)**	**34 (8.7)**	43 (4.5)
Semantic processing			
BPVS (/150)	**119 (35)**	**76 (56)**^b^	147 (2.1)
GNT (/30)	**11 (9.3)**	**3.4 (5.8)**^b^	26 (3.0)
Executive function			
D-KEFS Stroop inhibition (sec)	**137 (98)**^b^	**108 (70)**	55 (13)
Digit span reverse (/12)	5.6 (2.9)	6.1 (3.2)	7.3 (1.9)
Social cognition — TASIT			
Emotion (/14)	**7.1 (2.9)**	**5.8 (2.8)**	11 (1.3)
Total sarcasm (/24)	**13 (6.7)**^a^	**11 (5.9)**	22 (2.3)
Simple sarcasm (/12)	**7.4 (3.5)**	**5 (3.5)**	10 (1.4)
Paradoxical sarcasm (/12)	**7 (2.9)**	**5.7 (2.9)**	11 (1.3)
Other skills			
Digit span forward (/12)	8.8 (1.6)	6.8 (2.6)	8.9 (1.7)
VOSP (/20)	16 (3.8)	15 (3.5)	18 (1.6)

Mean (standard deviation) values shown; maximum scores are shown in parentheses after names of tests. Significant group differences in t tests p < 0.05 relative to the healthy control group are shown in bold. BPVS, British Picture Vocabulary Scale; bvFTD, behavioural variant frontotemporal dementia; D-KEFS, Delis–Kaplan Executive Function System; GNT, Graded Naming Test; n/a, not available; NART, National Adult Reading Test; RMT, Recognition Memory Test; sec, seconds; svPPA, semantic variant primary progressive aphasia; TASIT, the Awareness of Social Inference Test; VOSP, Visual Object and Space Perception; WASI, Wechsler Abbreviated Scale of Intelligence (see also Supplementary material on-line).

a Data from 27 patients (two unable to understand task).

b Significant difference between bvFTD and svPPA patients.

**Table 2:** Summary of DTI correlations with emotion identification in patient groups.

Contrast	Cluster no.	Voxels	x	y	z	p-Value	White matter tract/region
*Increased axial diffusivity*							
All patients	1	58,082	3	−4	10	<0.001	Fornix
bvFTD only	1	50,442	2	2	15	<0.001	Fornix
svPPA only	1	27,988	12	−7	3	0.002	Right anterior thalamic radiation
	2	2852	−38	1	27	0.02	Left superior frontal lobe^a^
	3	36	37	−79	−2	0.05	Right inferior occipital lobe^a^
	4	33	40	−68	−4	0.05	Right inferior occipital lobe^a^

*Increased radial diffusivity*							
All patients	1	68,045	3	−10	11	< 0.001	Right anterior thalamic radiation
bvFTD only	1	45,523	2	4	16	<0.001	Fornix
svPPA only	1	48,721	4	−11	9	0.001	Right anterior thalamic radiation
bvFTD > svPPA^b^	1	443	22	−6	14	0.04	Right anterior thalamic radiation
	2	361	46	−10	−22	0.05	Right inferior longitudinal fasciculus
	3	82	32	−31	9	0.05	Right inferior fronto-occipital fasciculus

*Increased trace diffusivity*							
All patients	1	63,092	3	−7	10	<0.001	Right anterior thalamic radiation
Decreased fractional anisotropy							
bvFTD only	1	1557	−3	29	5	0.02	Genu corpus callosum

Diffusion tensor tractography (DTI) associations of altered white matter diffusivity metrics associated with TASIT emotion identification score are summarised. Cluster numbering indexes statistically independent anatomical associations within each contrast generated using the FSL cluster command. Results are corrected for multiple comparisons at whole brain level using family-wise error correction (p = 0.05) and ordered by statistical significance and size (number of voxels in the cluster). Peak co-ordinates and anatomical associations are based on centre-of-gravity of cluster and are displayed in MNI standard space. bvFTD, behavioural variant frontotemporal dementia; svPPA, semantic variant primary progressive aphasia.

a Cluster peak co-ordinates not within JHU ICBM-DTI-81 white-matter atlas (white matter region assigned on visual inspection; other coordinates within white matter tract identified using JHU ICBM-DTI-81 white-matter atlas).

b Note here and in Fig. 1, this group interaction contrast is based on an *inverse* correlation with RD.

**Table 3:** Summary of DTI correlations with sarcasm identification in patient groups.

Contrast	Cluster no.	Voxels	x	y	z	p-Value	White matter tract/region
*Increased axial diffusivity*							
All patients	1	10,986	33	2	−7	0.005	Right uncinate fasciculus
bvFTD only	1	23,527	11	10	0	0.004	Right anterior thalamic radiation
	2	565	32	−49	38	0.05	Right superior parietal lobe^a^
	3	247	14	−67	48	0.05	Right superior parietal lobe^a^
	4	111	36	−62	40	0.05	Right superior parietal lobe^a^
	5	92	36	−65	31	0.05	Right parieto-occipital lobe^a^
	6	76	−56	−29	−10	0.05	Right superior longitudinal fasciculus: temporal part
	7	29	23	−55	53	0.05	Right superior parietal lobe^a^
svPPA only	1	389	48	−12	−22	0.04	Right inferior longitudinal fasciculus

*Increased radial diffusivity*							
All patients	1	9125	33	3	−8	0.009	Right uncinate fasciculus
	2	1183	47	−47	2	0.03	Right superior longitudinal fasciculus: posterior part
bvFTD only	1	4686	24	24	−5	0.02	Right uncinate fasciculus
	2	3209	43	−16	−18	0.03	Right inferior longitudinal fasciculus
	3	1127	−16	29	−12	0.04	Left anterior corona radiata
	4	56	45	−54	−8	0.05	Right inferior temporal lobe^a^
svPPA only	1	156	53	−11	−22	0.05	Right inferior longitudinal fasciculus

*Increased trace diffusivity*							
All patients	1	9436	33	4	−8	0.007	Right uncinate fasciculus
	2	42	53	−48	6	0.05	Right superior longitudinal fasciculus: posterior part
bvFTD only	1	9436	32	9	−8	0.02	Right uncinate fasciculus
	2	869	−13	30	−12	0.04	Left anterior corona radiata
	3	145	−26	23	−14	0.05	Left uncinate fasciculus
svPPA only	1	154	53	−10	−22	0.05	Right inferior longitudinal fasciculus

Diffusion tensor tractography (DTI) associations of altered white matter diffusivity metrics associated with TASIT total sarcasm identification score are summarised. Cluster numbering indexes statistically independent anatomical associations within each contrast generated using the FSL cluster command. Results are corrected for multiple comparisons at whole brain level using family-wise error correction (p = 0.05) and ordered by statistical significance and size (number of voxels in the cluster). Peak co-ordinates and anatomical associations are based on centre-of-gravity of cluster and are displayed in MNI standard space.

a Cluster peak co-ordinates not within JHU ICBM-DTI-81 white-matter atlas (white matter region assigned on visual inspection; other coordinates within white matter tract identified using JHU ICBM-DTI-81 white-matter atlas); bvFTD, behavioural variant frontotemporal dementia; svPPA, semantic variant primary progressive aphasia.

**Table 4:** Summary of VBM correlates of sarcasm identification in patient groups.

Behavioural Correlate	Brain region	Cerebral hemisphere	Cluster size (voxels)	p value
Combined groups	Anterior TL, OFC	R	107,453	0.02
bvFTD only	Anterior TL, OFC	Bilateral	318,805	0.01
bvFTD > svPPA	Parahippocampal, fusiform gyri	L	61,222	0.03

Voxel-based morphometry (VBM) associations of TASIT total sarcasm identification score are summarised. All correlations shown are positive and significant after family-wise error correction for multiple comparisons at whole brain level (p < 0.05). bvFTD, behavioural variant frontotemporal dementia; L, left hemisphere; OFC, orbitofrontal cortex; R, right hemisphere; svPPA; semantic variant primary progressive aphasia; TL, temporal lobe.

## Supplementary data

Supplementary material Details of neuropsychological tests and disease maps.
