# Supplementary material for: White matter tract signatures of impaired social cognition in frontotemporal lobar degeneration
Source: Neuroimage Clin. 2015 Jun 23;8:640–51. doi: 10.1016/j.nicl.2015.06.005 (PMC4513187; doi:10.1016/j.nicl.2015.06.005)
Supplement: Supplementary material — Details of neuropsychological tests and disease maps. [file mmc1.docx]

## SUPPLEMENTARY MATERIAL. White matter tract signatures of impaired social cognition in frontotemporal lobar degeneration, by LE Downey et al

**Description of general neuropsychological tests**

The Wechsler Abbreviated Scale of Intelligence (Wechsler, 1999) is an abbreviated version of the Wechsler Adult Intelligence Scale, yielding a global measure of intelligence represented by a verbal and performance IQ score. The Recognition Memory Test for faces (Warrington, 1984) presents 50 faces with an orienting question. The participant is then presented with the target paired with an unfamiliar face and asked to judge which they have seen before. The same procedure is used for the Recognition Memory Test for words. The British Picture Vocabulary Scale (Dunn, Dunn & Whetton, 1982) tests comprehension with a non-verbal output. Participants must match one of four pictures to a target word of increasing difficulty. The Graded Naming Test (McKenna & Warrington, 1983) requires participants to name line drawings that become increasingly less common. For the Object Decision subtest of the Visual Object and Space Perception battery (Warrington & James, 1991) participants are asked to identify the silhouette of a 75 degree rotated real object from three nonsense silhouettes of similar complexity. Digit Span task (Wechsler, 1987) was used to assess working memory by repeating number strings of increasing length. The D-KEFS Colour Word Interference Test (Delis, Kaplan & Kramer, 2001) is an adaptation of the Stroop test, where colour words are presented in a conflicting ink colour. Here participants are timed naming the colour of the ink for 50 of these words.

Wechsler, D., Wechsler Abbreviated Scale of Intelligence. San Antonio: Pearson; 1999.

Warrington, E. K., 1984. *Recognition Memory Test: Manual*. Berkshire UK: NFER-Nelson.

Dunn, L. M., Dunn, L. M. & Whetton, V., 1982. *British Picture Vocabulary Scale – Revised*. Windsor: UK. NFER-Nelson.

McKenna, P., & Warrington, E.K., 1983. *The Graded Naming Test.* Windsor, Berks: NFER-Nelson.

Warrington, E. K., & James, M., 1991. *The Visual Object and Space Perception Battery (VOSP)*. Bury St. Edmunds, England: Thames Valley Test Co.

Wechsler D. Wechsler memory scale - revised. San Antonio: The Psychological Corporation; 1987.

Delis, D. C., Kaplan, E., & Kramer, J. H., 2001. *Delis–Kaplan Executive Function System (D-KEFS): Examiner’s manual*. San Antonio, TX: The Psychological Corporation.

**Figure S1**


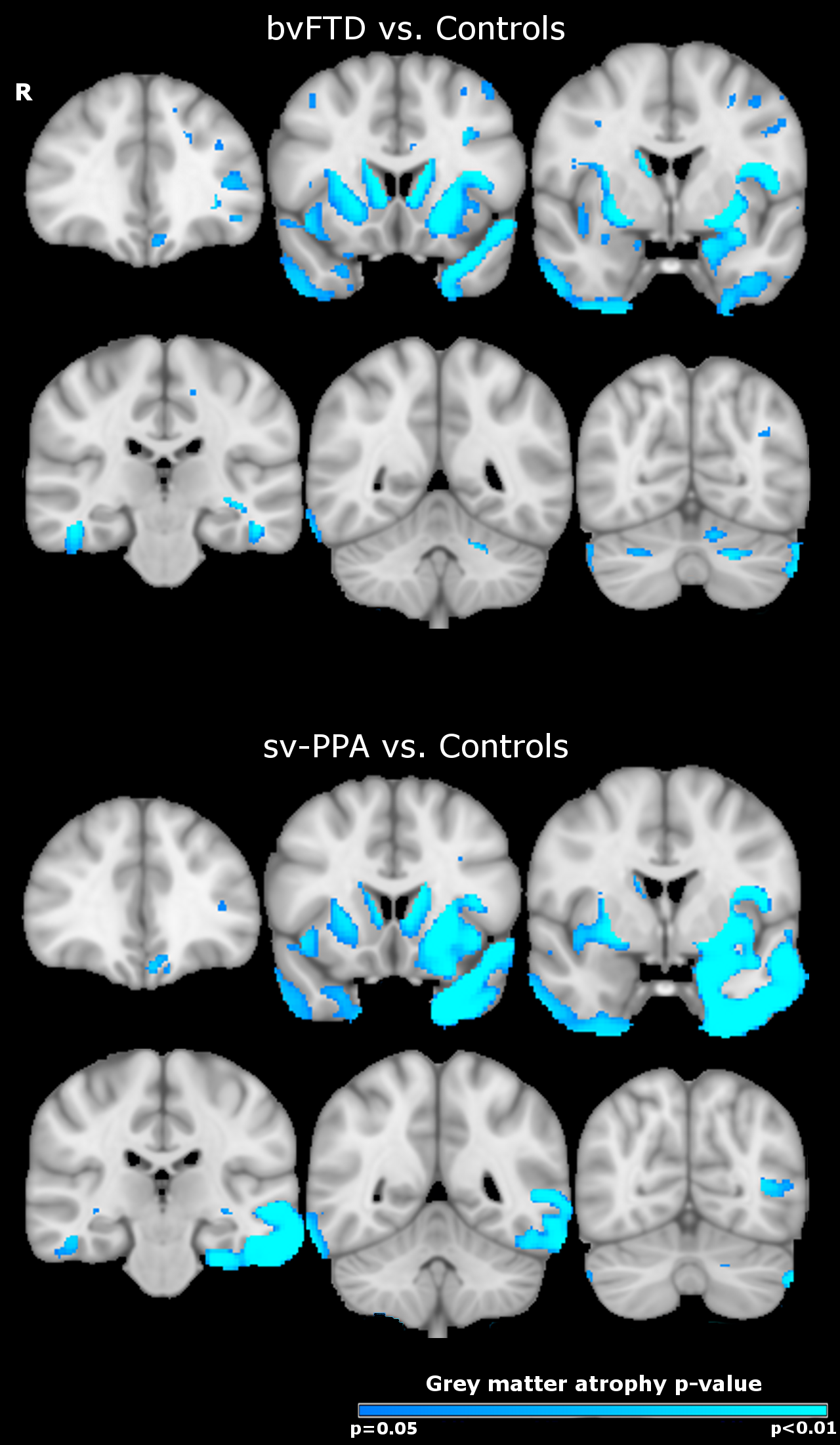


Distribution of grey matter atrophy in bvFTD (above) and svPPA (below) when compared with healthy controls. In this VBM analysis, each patient group was compared with a separate historical group of 20 healthy individuals for which brain MR images were previously obtained using the same scanner and acquisition parameters and covarying for age and TIV. Results are overlaid on a coronal MNI152 template brain and are uncorrected at p<0.05 for display purposes; the right hemisphere (R) is displayed on the left. The colour scale indexes uncorrected p-values in order to indicate liberally disease extent. Key: bvFTD, behavioural variant frontotemporal dementia; svPPA, semantic variant of primary progressive aphasia.

**Figure S2**


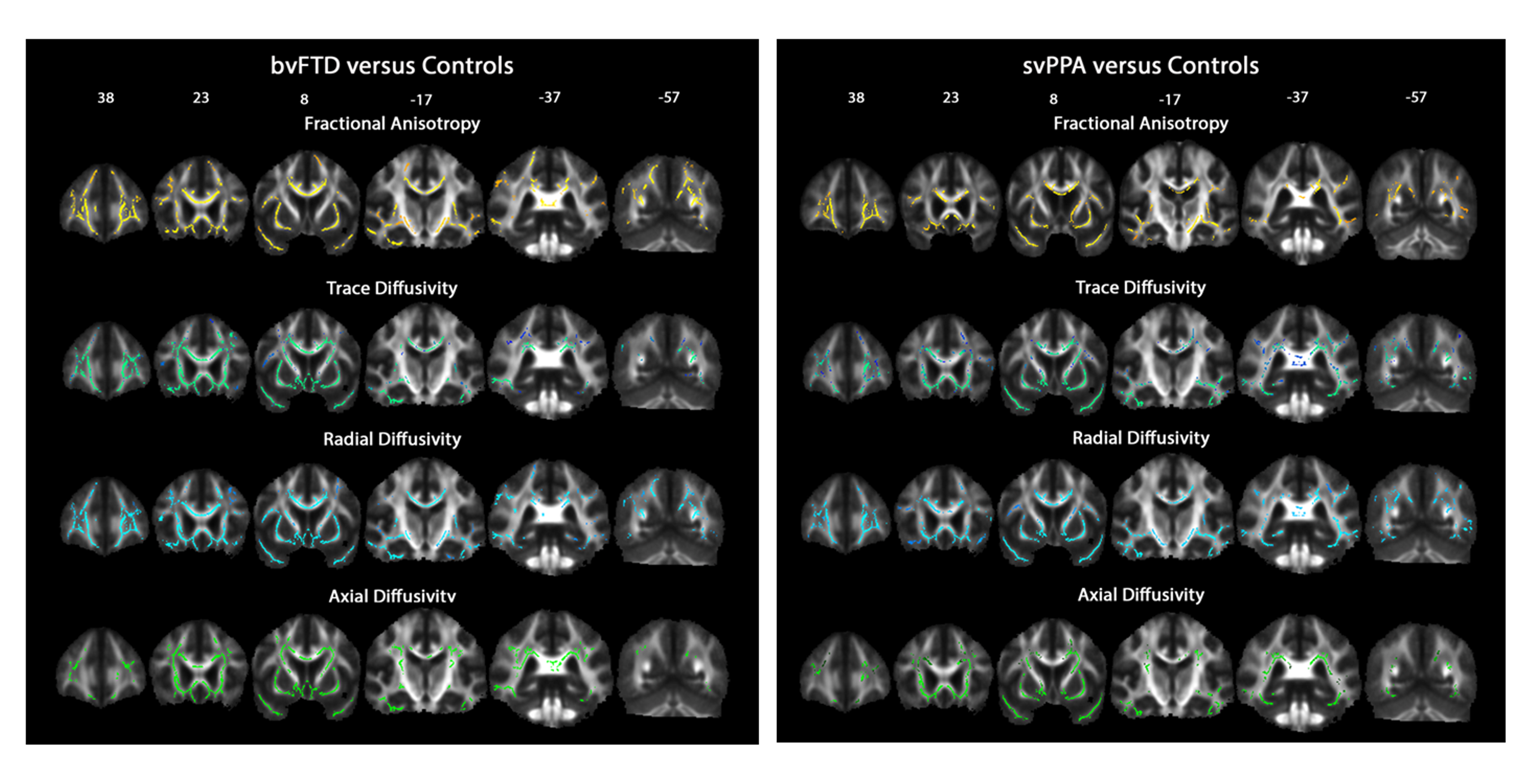


Distribution of white matter tract alterations in bvFTD (left panels) and svPPA (right panels) compared with healthy controls. In this TBSS analysis, each patient group was compared with the age- and gender-matched historical group used in the VBM analysis. Results are overlaid on coronal sections of a customised group template brain image in MNI standard space, after family-wise error correction at p<0.05 over the whole brain (yellow-orange codes decreased fractional anisotropy, blue-green codes increased diffusivity, brighter colours code higher p-values); the right hemisphere is displayed on the left and the y-coordinate (mm) of the plane of each section is indicated. Key: bvFTD, behavioural variant frontotemporal dementia; svPPA, semantic variant of primary progressive aphasia.
